# Simultaneous CRISPR/Cas9-induced double-strand breaks are lethal in models of pancreatic cancer

**DOI:** 10.1172/JCI190121

**Published:** 2026-05-15

**Authors:** Selina Shiqing K. Teh, Akhil Kotwal, Alexis Bennett, Eitan Halper-Stromberg, Laura Morsberger, Saum Zamani, Yanan Shi, Alyza Skaist, Qingfeng Zhu, Kirsten Bowland, Hong Liang, Ralph H. Hruban, Chien-Fu Hung, Robert A. Anders, Nicholas J. Roberts, Robert B. Scharpf, Michael Goldstein, Ying S. Zou, James R. Eshleman

**Affiliations:** 1 Department of Pathology, The Sol Goldman Pancreatic Cancer Research Center;; 2 Department of Radiation Oncology and Molecular Radiation Sciences; and; 3 Department of Oncology, The Johns Hopkins University School of Medicine, Baltimore, Maryland, USA.

**Keywords:** Genetics, Oncology, Cancer gene therapy, Genetic instability, Radiation therapy

## Abstract

While radiation is an effective oncologic therapy, killing cancer by inducing DNA double-strand breaks (DSBs), it lacks specificity for neoplastic cells. We have previously adapted the CRISPR/Cas9 gene-editing technology as a cancer-specific treatment modality targeting somatic mutations in pancreatic cancer (PC). However, its tumoricidal potential remains unclear, especially in comparison with therapeutic doses of radiation. Here, we demonstrate that CRISPR/Cas9-induced DSBs are more cytotoxic in PCs than a comparable number of radiation-induced DSBs. We observed more than 90% tumor growth inhibition by targeting 9 sites with cancer-specific sgRNAs. Through both bioinformatics and cytogenetics analyses, we found that CRISPR/Cas9-induced DSBs triggered ongoing chromosomal rearrangements, with 87% of structural variants not directly produced from the initial CRISPR/Cas9-induced DSBs, and chromosomal instability peaking before cell death. By comparing the cytotoxicity of CRISPR/Cas9- and radiation-induced DSBs, we demonstrated that the number of DSBs required to achieve equitoxic effects was approximately 3 times higher for radiation than CRISPR/Cas9. Finally, we showed that PC cells that had survived CRISPR/Cas9 targeting retained susceptibility to subsequent CRISPR/Cas9-induced DSBs at different genomic sites with more than 87% growth inhibition. Together, our data support the therapeutic potential of CRISPR/Cas9 as an anticancer strategy.

## Abstract 2

The CRISPR-Cas9 system can be adapted into a cancer killing strategy by inducing precise and unrecoverable DNA damage.

## Graphical abstract

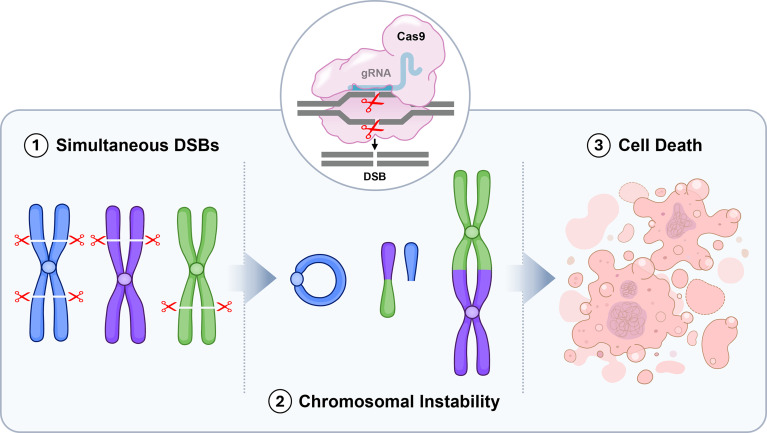

## Introduction

Chromosomal instability (CIN), a cancer hallmark, produces numerical or structural chromosomal abnormalities. Under selective pressure, such as tumor evolution and therapeutic intervention, CIN enables clonal evolution of heterogeneous karyotypes and phenotypic adaptation, promoting drug-resistant clones, bypassing oncogene addiction, and expanding oncogene-independent subclones (1–3). However, CIN carries a fitness cost: many karyotypes are nonviable, and extreme CIN, intolerable to cancer cells, correlates with better prognosis (4, 5). Elevated chromosome mis-segregation that drives high CIN can suppress tumor growth (6–8), suggesting that for tumors to propagate sustainably, the equilibrium between the tumor-promoting CIN and tumor-suppressive CIN has to be optimal and that strategies to shift the equilibrium toward cancer cell killing could be leveraged to improve patient outcomes (1).

Double-strand DNA breaks (DSBs) are known to be the most lethal of all DNA lesions (9, 10). This cytotoxicity originates from the DNA damage response that either repairs DSBs or triggers senescence or cell death, and many conventional anticancer strategies, such as radiation therapy and some chemotherapies, exploit it for cancer killing (9). Despite multiple repair pathways, DSBs remain dangerous to cell survival as they directly disrupt the genome integrity, and erroneous repair can fuel CIN (9–11). One such consequence is the initiation of breakage-fusion-bridge cycles, in which broken chromosomes fuse to form dicentrics, anaphase breaks, structural variants (SVs), and telomere-free chromosome ends, propagating further genomic rearrangements (12–14).

Studies using endonucleases, such as I-*Sce*I and CRISPR/Cas9 that can induce DSBs at specific locations, have shown a positive correlation between increased number of DSBs and growth inhibition (15–17). CRISPR/Cas9-induced DSBs have been linked to chromothripsis (15), chromosomal rearrangements (18, 19), and cytotoxicity (20–22). However, most studies seek to minimize cytotoxicity to improve editing (22–24), with very few exploiting this cytotoxicity for cell killing (20, 25, 26). Recent studies have also reported upregulation of the p53 pathway in Cas9-expressing cells to inhibit genetic perturbations, indicating *TP53* status must be considered when assessing CRISPR/Cas9-induced cytotoxicity (27–29).

We present evidence that CRISPR/Cas9 is a potent anticancer strategy for pancreatic cancer (PC). PC is the third leading cause of cancer death in the United States, with a dismal 5-year survival rate of 13% (30). More than 90% of PCs are pancreatic ductal adenocarcinomas (PDACs) (31), and approximately 70% of PDACs harbor *TP53* inactivation (32). While radiation therapy damages DNA directly or indirectly via free radicals (33), we find CRISPR/Cas9-induced DSBs are more cytotoxic than clinically relevant doses of radiation, driving extreme CIN and cell death. More broadly, CRISPR/Cas9 may serve as a selective, highly cytotoxic approach against cancers.

## Results

### CRISPR/Cas9-induced DSBs inhibit cancer cell growth.

We designed multitarget sgRNAs with 2–16 target sites in noncoding regions of the human genome to avoid confounding cytotoxicity caused by gene essentiality (Supplemental Table 1; supplemental material available online with this article; https://doi.org/10.1172/JCI190121DS1; sgRNA design previously described in ref. 26). Negative controls included 2 nontargeting sgRNAs (NT1 and NT2), while positive controls included 3 sgRNAs targeting repetitive elements. An *HPRT1*-targeting sgRNA was also designed to validate Cas9 activity via 6-thioguanine resistance. The number of predicted target sites of each sgRNA is noted in parentheses: e.g., 52F(3) has 3 sites; “(rep)” denotes targets in repetitive elements. We generated Cas9-expressing cell lines from 2 *TP53*-inactivated PC lines, Panc10.05 (homozygous I255N) and TS0111 (homozygous C275Y), and documented Cas9 activity (Supplemental Figure 1A) using a previously described assay (26). Transduction of negative and positive control sgRNAs into parental, dead Cas9–expressing, and Cas9-expressing cells showed growth inhibition only in Cas9-expressing cells with positive control, repetitive element–targeting sgRNAs, indicating multiple DSBs are required for growth inhibition (Supplemental Figure 1B).

**Figure 1:**
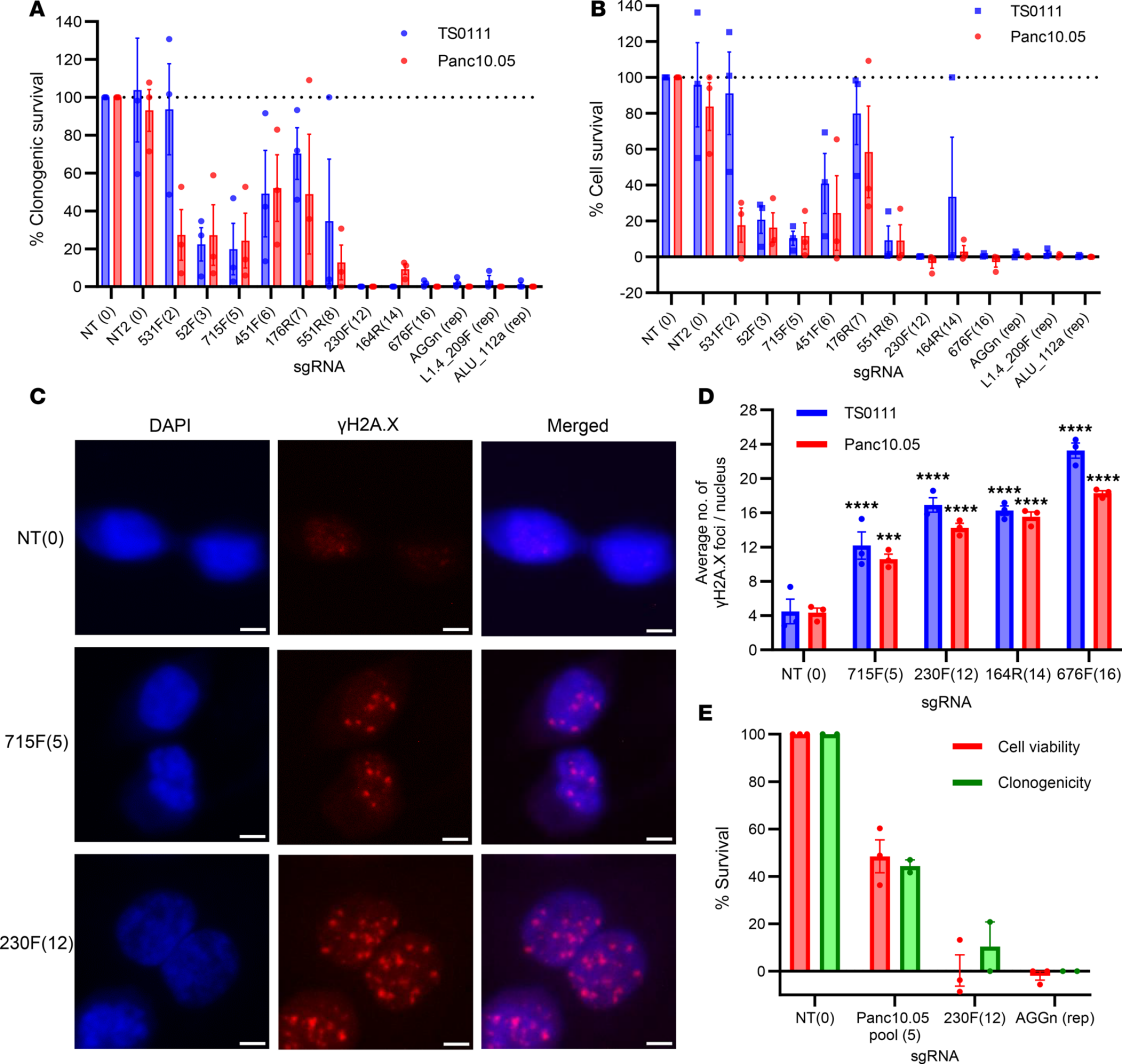
Increased CRISPR/Cas9-induced DSBs inhibit cancer cell growth. (**A**) Clonogenic survival with increased number of CRISPR/Cas9 target sites in the human genome of 2 PC cell lines. Number of target sites in parentheses; “rep” indicates repetitive element-targeting. *N* = 3; mean ± SEM, normalized to NT. (**B**) Cell survival with increased number of CRISPR/Cas9 target sites as detected by alamar blue cell viability assay. *N* = 3; mean ± SEM, normalized to NT. (**C**) Representative images of γH2A.X staining in Panc10.05 cells transduced with NT or 715F(5) or 230F(12) multitarget sgRNAs. Images at 40× original magnification; scale bar is 5 μm. *N* = 3. (**D**) Number of γH2A.X foci as a function of the number of CRISPR/Cas9 target sites. >100 nuclei were analyzed for each condition. Dunnett’s test between NT and each multitarget sgRNA; ***P < 0.001, ****P < 0.0001. *N* = 3; mean ± SEM. (**E**) Clonogenic and cell survival 21 days after electroporating in CRISPR/Cas9 with multitarget sgRNAs or a pool of 5 sgRNAs targeting different noncoding mutations in the Panc10.05 genome. *N* = 2/3; mean ± SEM, normalized to NT.

To test the hypothesis that growth inhibition increased with the number of simultaneously induced DSBs, we transduced multitarget sgRNAs into Cas9-expressing cells and performed clonogenicity and cell viability assays, ending when NT controls reached full confluence (1–2 months). We found that in general, clonogenic and cell survival decreased as a function of the number of sgRNA target sites (Figure 1, A and B). Multitarget sgRNAs with 12 target sites or more consistently produced >90% clonogenic inhibition, with 230F(12) demonstrating cytotoxicity similar to that of positive control sgRNAs. However, some variability was observed; for example, 451F(6) and 176R(7) sgRNAs were less inhibitory than 715F(5) sgRNA. Significant decrease in clonogenic inhibition was detected in only 1 of the cell lines treated with the 531F(2) sgRNA (Dunn-Šidák test, *P* = 0.040).

We performed γH2A.X staining to quantify CRISPR/Cas9-induced DSBs following multitarget sgRNA transduction. Given the hypodiploidy of Panc10.05 (39–40 chromosomes) and the hyperdiploidy of TS0111 (50–61 chromosomes), we expected more foci in TS0111 at the same target sites. We found that an increased number of sgRNA target sites correlated with an increased number of γH2A.X foci in both cell lines 48 hours posttransduction, with TS0111 cells generally exhibiting more foci (Figure 1, C and D, and Supplemental Figure 1C). Transduction of ALU_112a resulted in an excessive number of γH2A.X foci that was uncountable (Supplemental Figure 1D), consistent with the fact that ALU_112a is expected to generate approximately 66,533 DSBs per cell (Cas-OFFinder, ref. 34). γH2A.X staining of AGGn (repetitive element-targeting sgRNA) at earlier time points also showed that foci were below saturation before 48 hours (Supplemental Figure 1E). The baseline foci number was ~4 (Figure 1D), consistent with previous literature that established that malignant cells tend to have elevated levels of γH2A.X at baseline (35, 36). Since the number of γH2A.X foci correlated with the number of CRISPR/Cas9 target sites and none of the cells transduced with multitarget sgRNAs showed anomalous foci formation, our data suggest that the clonogenic inhibition by multitarget sgRNAs was a result of targeted DSB induction and not widespread off-target activities.

To ensure that our observations were not limited to constitutive expression of CRISPR/Cas9, we electroporated CRISPR/Cas9 ribonucleoprotein (RNP) complex into both cell lines for transient expression. We introduced NT, 230F(12), and AGGn sgRNAs, and for Panc10.05, a combination of 5 sgRNAs targeting 5 Panc10.05-specific noncoding mutations (Panc10.05 pool; designed using a protospacer adjacent motif–based [PAM-based], cancer-specific approach previously described in ref. 26) (Supplemental Table 2), into cells. We performed a clonogenic survival assay for 21 days, instead of 1–2 months as in our previous assays. Our results were similar to our data in Figure 1, A and B, in which 230F(12) showed >90% clonogenic inhibition (Figure 1E and Supplemental Figure 2A). Differences could be attributed to the confounding effect of cells that did not receive the CRISPR/Cas9 RNPs and survived. Interestingly, the Panc10.05-specific sgRNAs (each at 1/5 of the concentration used for multitarget sgRNAs) yielded >50% growth inhibition (Figure 1E), showing that transient expression of these mutation-targeting sgRNAs can inhibit PC cell growth.

The cell line–specific sgRNAs target somatic, noncoding mutations in cancer cells that are absent in patient-matched normal cells (26). To study the impact of these cancer-specific sgRNAs on normal cells, we treated 2 primary skin fibroblast and 2 cancer-associated fibroblast cell lines, all derived from patients, with a pool of 9 sgRNAs specific to either TS0111 or Panc10.05; cultured for 3–4 weeks; and assessed cell survival (Supplemental Figure 2B and Supplemental Tables 2 and 3). No significant differences versus negative controls were observed, indicating these cancer-specific sgRNAs do not inhibit growth of normal cells lacking the targets.

### Simultaneous CRISPR/Cas9 targeting inhibits tumor and metastatic growth.

**Figure 2:**
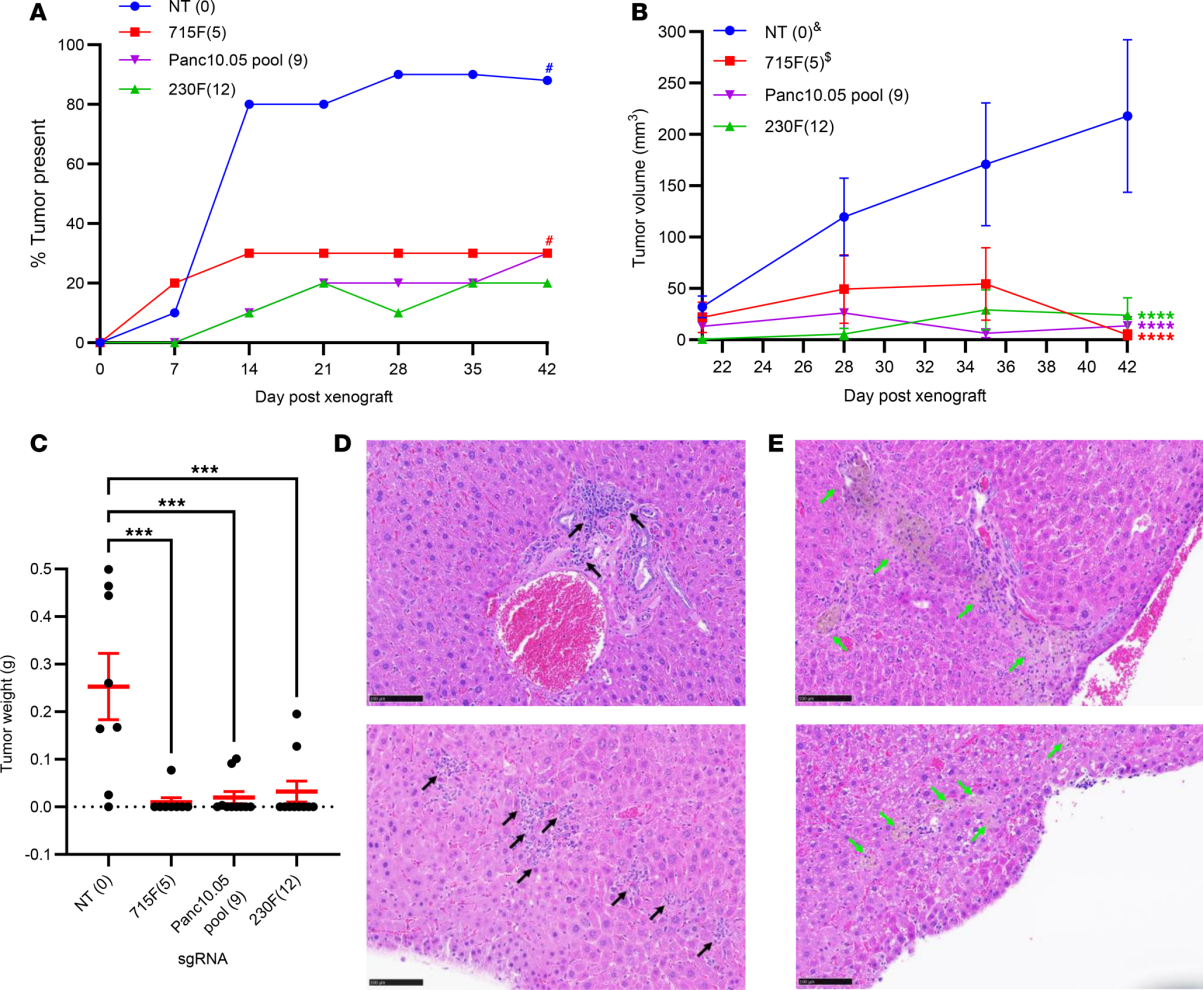
Simultaneous CRISPR/Cas9 targeting inhibits tumor growth. (**A**–**C**) Tumor growth experiment in subcutaneous xenograft models. Panc10.05 Cas9-expressing cells transduced with the following sgRNAs: NT, 715F(5), 230F(12), or a pool of 9 sgRNAs targeting different noncoding mutations unique to Panc10.05 (Panc10.05 pool) were injected into nude mice for tumor growth. (**A**) Percentage of tumors present postxenograft. # indicates absence of 2 data points due to early death around week 5 (33–36 days). (**B**) Tumor volume measurements postxenograft. Dunn-Šidák test between NT and the other treatment groups on week 6, all ****P < 0.0001. *N* = 10; mean ± SEM. & indicates absence of week 5 and 6 data points of 2 tumors due to early death. $ indicates absence of 2 data points from week 6 due to early death. (**C**) Tumor weight measurements on week 6 postxenograft. Dunnett’s test between NT (*N* = 8) and 715F(5): *P* = 0.0003 (*N* = 8), 230F(12): *P* = 0.0008 (*N* = 10), and Panc10.05 pool: *P* = 0.0004 (*N* = 10). ***P < 0.001. Mean ± SEM was shown. (**D** and **E**) Metastatic growth experiment in hemispleen injection mouse models of liver metastasis. Hematoxylin and eosin (H&E) staining of the liver sections of mice treated with (**D**) NT (*N* = 7) or (**E**) 230F(12) (*N* = 5) sgRNA-expressing PC cells. Black arrow: tumor growth; green arrow: tumor regression. The top and bottom panels represent liver sections from 2 different mice of the same treatment group. Images at 20× original magnification; scale bar is 100 μm.

To investigate whether increasing CRISPR/Cas9-induced DSBs would reduce tumor growth, we first transduced Panc10.05 Cas9-expressing cells with NT, 715F(5), 230F(12), ALU_112a, or the 9-sgRNA Panc10.05 pool (as in Supplemental Figure 2B and Supplemental Table 2). After puromycin selection of transduced cells, we injected them subcutaneously into both flanks of nude mice and monitored them for 6 weeks. The percentage of NT tumors present postxenograft peaked at 90%, followed by 30% of 715F(5) and Panc10.05 pool tumors and 20% of 230F(12) tumors (Figure 2A); ALU_112a yielded no tumors (data not shown). Significant decreases in tumor volumes between NT and 230F(12) or Panc10.05 pool were observed as early as week 4 [Figure 2B; Dunn-Šidák test, NT and 230F(12): *P* = 0.010, NT and Panc10.05 pool: *P* = 0.043, all *N* = 10]. By week 6, significant decreases in tumor volumes between NT and the remaining treatment groups were observed (Figure 2B, all *P* < 0.0001). Tumors were harvested and weighed by the end of week 6 (Figure 2C and Supplemental Figure 2C), which showed significant decreases in tumor weight between NT and the rest of the treatment groups (all *P* < 0.0001) but no differences in body weight (Supplemental Figure 2D). To determine whether CRISPR/Cas9 activity had occurred in tumors harvested, we extracted genomic DNA from the 2 outlier 230F(12) tumors, PCR-amplified the 230F(12) target regions, and performed next-generation sequencing (NGS). We found that the mutation frequency was 0.97% and 0.64% in 230F(12) tumors, suggesting insufficient CRISPR/Cas9 activity enabled tumor growth.

Since the primary site of PC metastasis is the liver, we established a hemispleen injection mouse model of liver metastasis using Panc10.05 Cas9-mNeonGreen–expressing cells (Supplemental Figure 2E). Following transduction and selection, NT- and 230F(12)-treated cells were injected into half of the spleen of nude mice during hemisplenectomy to seed the liver. At 30 days after surgery, livers were harvested, sectioned, and stained with hematoxylin and eosin (H&E) for histology analysis (Figure 2, D and E). While we observed PC metastases in NT-treated livers (Figure 2D; black arrows), tumor regression was detected in the livers of 230F(12)-treated cells (Figure 2E; green arrows), showing that multitargeting by CRISPR/Cas9 suppresses liver metastases.

We subsequently built a doxycycline-inducible Cas9 (Dox-iCas9) system expressing Cas9 and EGFP (doxycycline-inducible) and a U6-driven sgRNA: NT, 230F(12), and L1.4_209F. We transduced Panc10.05 with the cloned vectors, generated derivative cell lines through puromycin selection, and injected them subcutaneously into nude mice. We also prepared a no–Dox-iCas9, sgRNA-only control, “230F(12) only,” in which no EGFP would be detected in the presence of doxycycline. After tumors formed, we started doxycycline hyclate feed while measuring tumor growth (Supplemental Figure 3A). By the end of week 3, tumors were harvested, weighed, and digested for flow cytometry to detect the presence of EGFP^+^ cells as an indicator of PC cells (Supplemental Figure 3, B–D). Although body weights, tumor volumes, and tumor weights among treatment groups did not differ significantly (Supplemental Figure 3, A–C), we found significant decreases in PC cells in digested tumors expressing 230F(12) and L1.4_209F compared with NT control [Supplemental Figure 3D; Dunnett’s test, NT and 230F(12): *P* = 0.0005, NT and L1.4_209F: *P* = 0.0007, all *N* = 3]. We hypothesized that the lack of phenotypic effect was due to the incomplete induction of Cas9, as our in vitro correlates (from the same pool of cells used for xenograft) showed a 79.6% induction of Cas9-EGFP in Cas9+NT while treated with doxycycline (Supplemental Figure 3E), suggesting that approximately 20% of transduced cells were not expressing Cas9. In vitro data also showed reduced growth inhibition of Cas9+230F(12), with 18.5% of living cells expressing EGFP, in contrast with prior near-complete clonogenic suppression in 230F(12)-treated cells, suggesting that reduced expression of Cas9 or sgRNA may be causing tumor growth. Our preliminary data suggest that multitargeting of CRISPR/Cas9 reduces cancer cells in established tumors, and further refinement of the Dox-iCas9 model is necessary to observe a meaningful phenotypic effect.

To assess delivery to liver metastases, we used the hemispleen model and performed hydrodynamic injection to deliver Firefly luciferase–expressing plasmid to mouse livers (Supplemental Figure 3F). After 72 hours, livers were dissociated and sorted into 2 cell populations, one for liver cells (Supplemental Figure 3G) and one for PC metastatic cells, based on the presence of mNeonGreen fluorescence (Supplemental Figure 3H). Intracellular staining and flow cytometry detected the presence of luciferase in 23.3% of metastatic PC cells but not in liver cells (Supplemental Figure 3, G and H), demonstrating feasibility of gene therapy delivery to PC liver metastases.

### Multitarget sgRNAs exhibit on-target and minimal off-target activities.

**Figure 3:**
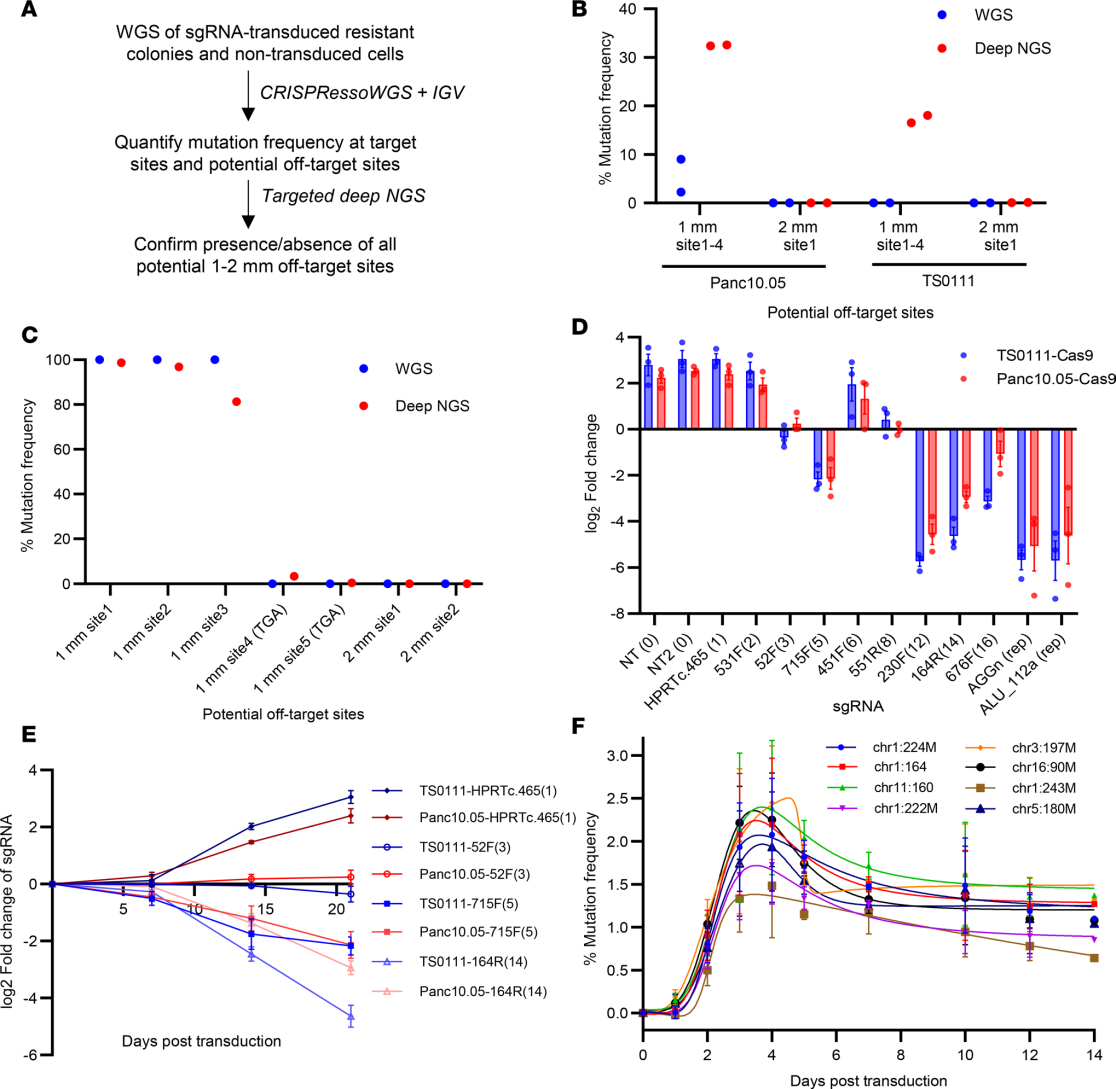
Multiple CRISPR/Cas9 scissions lead to delayed cell death. (**A**) Analysis workflow for quantification of on- and off-target sites in resistant colonies from clonogenicity assays. (**B** and **C**) Comparisons of mutation frequency at 1–2 mismatch (mm) sites detected by WGS and targeted deep NGS in resistant colonies. (**B**) 531F(2) sgRNA–resistant colonies. Four 1 mm sites were sequenced using the same primers. *N* = 2, mean ± SEM. (**C**) Panc10.05 164R(14) sgRNA–resistant colony. Noncanonical PAMs were indicated in parentheses. *N* = 1. (**D**) Fold-change of multitarget sgRNAs in 2 PC cell lines 21 days after transduction. Number of target sites in parentheses; “rep” indicates repetitive element targeting. *N* = 3; mean ± SEM. (**E**) sgRNA tag survival over time. *N* = 3; mean ± SEM. (**F**) Mutation frequencies of eight 164R(14) sgRNA target sites in Panc10.05 Cas9-expressing cells at various time points. *N* = 3; mean ± SEM. Bell-shaped least squares regression; *R*^2^ = 0.60–0.74. Relatively low percentages were due to the absence of antibiotic selection of transduced cells.

We subcultured surviving/resistant colonies from clonogenicity assays for an additional month before extracting genomic DNA to assess the targeting activity of the multitarget sgRNAs. Notably, we failed to obtain colonies from the 230F(12)- and 676F(16)-treated Panc10.05 cells, as well as from the 551R(8)-, 230F(12)-, and 164R(14)-treated TS0111 cells in all replicates (Table 1), indicating substantial cytotoxicity from these multitarget sgRNAs. We conducted WGS to evaluate predicted on-target and potential off-target sites (Figure 3A). Potential off-target sites with 1–4 base pair (bp) mismatches (mm) were predicted by CRISPOR (37), while off-target hits with gaps were identified using the Integrated DNA Technologies gRNA design checker (38). Through both manual inspection using the Integrative Genomics Viewer (IGV) and bioinformatics analyses, we found that more than 95% of mutations originated from on-target sites, and only 28% of 1 mm sites exhibited mutations among all potential off-targets (Table 1 and Supplemental Data 1). We did not detect mutations at off-target sites containing gaps in Panc10.05 colonies (data not shown). To validate these findings, we performed targeted deep sequencing (50,000× coverage) on all potential 1 mm and 2 mm sites (Figure 3A), revealing mutations exclusively in 1 mm sites, not in 2 mm sites (Figure 3, B and C, and Supplemental Figure 4, A and B). Our data demonstrate that deep NGS can reveal mutations that WGS cannot (Figure 3, B and C), likely due to the lower limit of detection by WGS (30× coverage). As an alternative approach to identify potential off-target sites, we analyzed all indels and SVs absent in the parental control, comparing sequence homology surrounding the mutations or breakpoints with the sgRNA sequence. None of the surrounding sequences had fewer than 5 mm compared with the original sgRNA (Supplemental Table 4). We also compared SVs in nontargeted regions among individual colonies transduced with the same sgRNAs and found that these SVs were unique to each colony (data not shown). We found 1 shared SV instance, but its breakpoint differed from the sgRNA sequence by 13 mm, suggesting it was likely present at a low level in the bulk cell line before selection by single-cell cloning.

We then compared the mutation frequencies of on-target sites across each colony to explore factors influencing variability in clonogenic inhibition (Figure 1A). Resistant colonies from 451F(6) and 176R(7) sgRNAs showed lower mutation frequencies of approximately 80% and approximately 36%, respectively, compared with 715F(5) (~97%). This suggests inadequate cutting activity at the 451F(6) and 176R(7) target sites, which contributed to lower clonogenic inhibition than 715F(5) (Supplemental Figure 4C). The 451F(6) sgRNA features a TT motif near the PAM, potentially resulting in reduced sgRNA expression when virally expressed (39). Additionally, single nucleotide variants (SNVs) were present in 4 of 6 on-target sites or PAMs of 451F(6) in Panc10.05, further affecting cutting efficiency (Supplemental Data 1). Similarly, SNVs were found in 4 of 7 on-target sites for 176R(7) in Panc10.05 and in 2 of 7 in TS0111, likely contributing to low overall mutation frequency and clonogenic inhibition (Supplemental Data 1). Furthermore, the average on-target mutation frequency of 531F(2) sgRNA–resistant colonies was higher in Panc10.05 (100%) than in TS0111 (40%; Supplemental Figure 4C), with mutation frequencies at 1 mm sites higher in Panc10.05 (Figure 3B). This indicates that decreased on- and off-target activities in TS0111 contributed to the observed differences in clonogenic inhibition between the 2 cell lines.

We quantified the copy number (CN) of each on- and off-target site in the surviving colonies to determine the number of DSBs induced by CRISPR/Cas9. For sgRNAs lacking a corresponding surviving colony, we estimated the total number of mutated sites based on CN at all on-target sites or by assuming that the mutated sites mirrored those of a different cell line with an available colony. We found that an increased number of predicted target sites generally correlated with a higher number of mutated sites (Supplemental Figure 4D). In Panc10.05, the total CN of target sites in the 52F(3), 715F(5), and 551R(8) colonies was comparable (9–10 cut sites), possibly explaining the similar clonogenic inhibition observed in Figure 1, A and B. The total CN of target sites also correlated with γH2A.X foci data (Supplemental Figure 4E; Pearson’s *r* for TS0111 = 0.90, *P* = 0.038; Panc10.05 = 0.98, *P* = 0.003), suggesting that most observed foci resulted from CRISPR/Cas9-induced DSBs.

### Simultaneous CRISPR/Cas9 targeting leads to delayed cell death.

As an independent measure of growth inhibition, we assessed sgRNA tag survival in the same cell lines, based on the premise that lethal sgRNAs would be eliminated from the sgRNA “tag” pool, while those with little or no cytotoxicity would be maintained or enriched. The 176R(7) sgRNA was excluded from subsequent analyses due to its low mutation prevalence in surviving colonies (Supplemental Figure 4C). We found that sgRNAs with a higher number of target sites exhibited greater sgRNA tag loss in Cas9-expressing cell lines but not in parental cell lines (lacking Cas9) 21 days posttransduction (Figure 3D and Supplemental Figure 5A). We compared the sgRNA tag survival results with the results obtained from the clonogenicity assays and found that the data are significantly correlated (Spearman’s *r* for Panc10.05 = –0.78, *P* = 0.001; TS0111 = –0.92, *P* < 0.0001), indicating that sgRNA tag survival is a reliable surrogate for clonogenicity assays. We also performed sgRNA tag survival in 4 additional PC cell lines, revealing a general inverse correlation between the number of sgRNA target sites and sgRNA fold-change, suggesting a consistent dose-response effect across 6 cell lines (Supplemental Figure 5B).

Interestingly, tag reduction for multitarget sgRNAs with a high number of cuts primarily occurred between days 7 and 21 posttransduction, rather than during the first 7 days (Figure 3E and Supplemental Figure 5, D and F). This was specific to Cas9-expressing cell lines, as parental cell lines showed no notable differences in sgRNA fold-changes across time points (Supplemental Figure 5, A, C, and E). A comparable experiment involving the introduction of Cas9 via electroporation for transient expression yielded similar results (Supplemental Figure 5G). To investigate whether the temporal delay in tag loss was due to delayed DSB induction, we transduced the 164R(14) sgRNA into Panc10.05 Cas9-expressing cells and measured mutation frequency at 8 on-target sites over 14 days. We found that scission and repair peaked at days 3–4 (Figure 3F, R^2^ = 0.60–0.74 for all 8 sites), consistent with prior studies (40, 41). Overall, our findings indicate that DSBs induced by CRISPR/Cas9 occur within the first few days but do not immediately trigger cell death, suggesting an alternative mechanism contributes to the delay in growth inhibition.

### Ongoing and peak CIN induced by CRISPR/Cas9 scissions.

**Figure 4:**
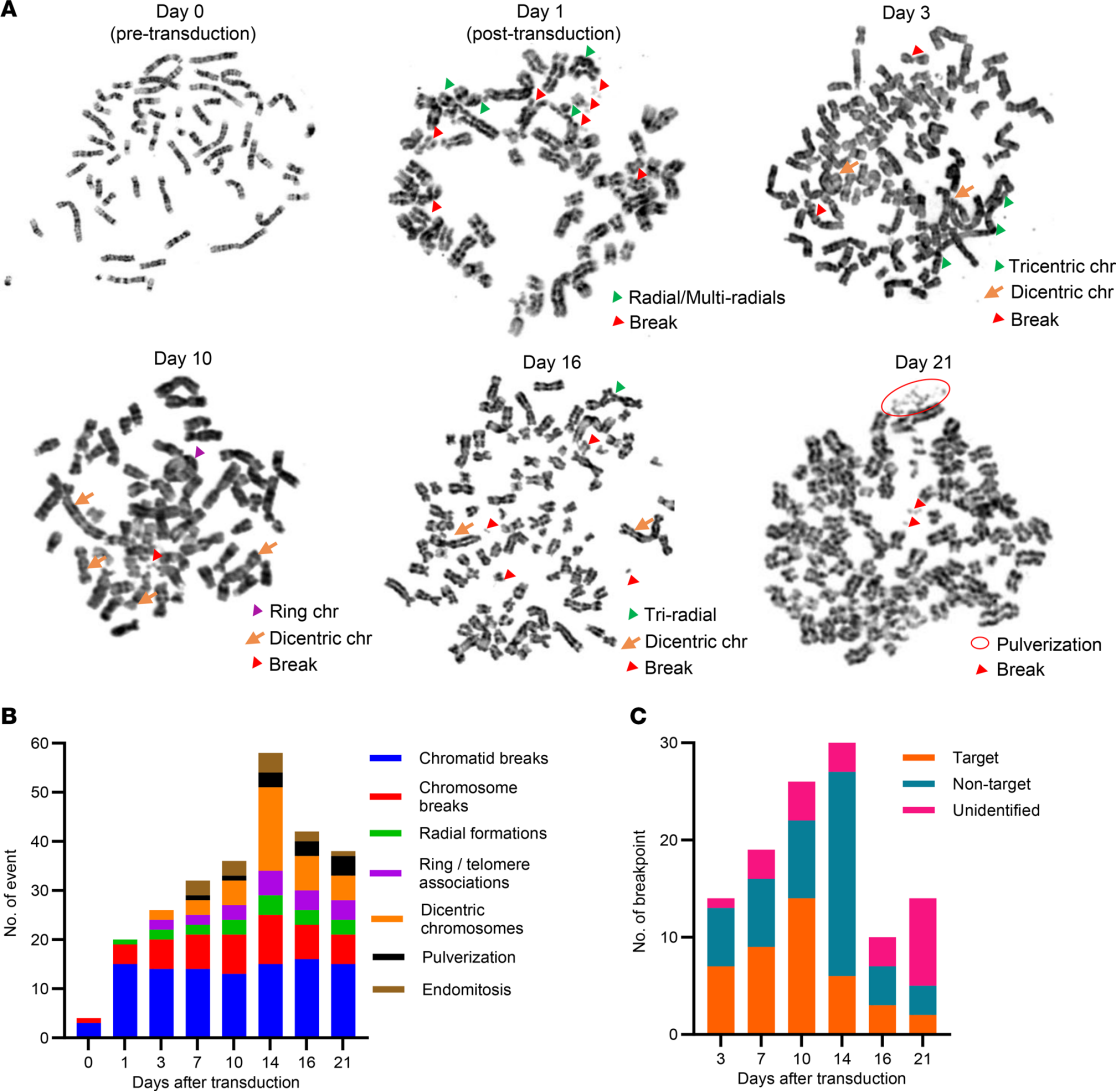
Ongoing CIN in multitarget sgRNA–transduced cells. (**A**–**C**) TS0111 Cas9-expressing cells were transduced with 164R(14) sgRNA and subjected to chromosome breakage assays. (**A**) Metaphase images of representative cells pre- and posttransduction of sgRNA. Karyotypic alterations are labeled. *N* = 1. (**B**) Cytogenetic changes (events per 100 metaphase cells) over time. (**C**) Quantification of breakpoints on dicentric, tricentric, and ring chromosomes, categorized by their chromosomal band locations to determine whether the breakpoint junction was located at 164R(14) sgRNA–targeted or nontargeted regions.

**Figure 5:**
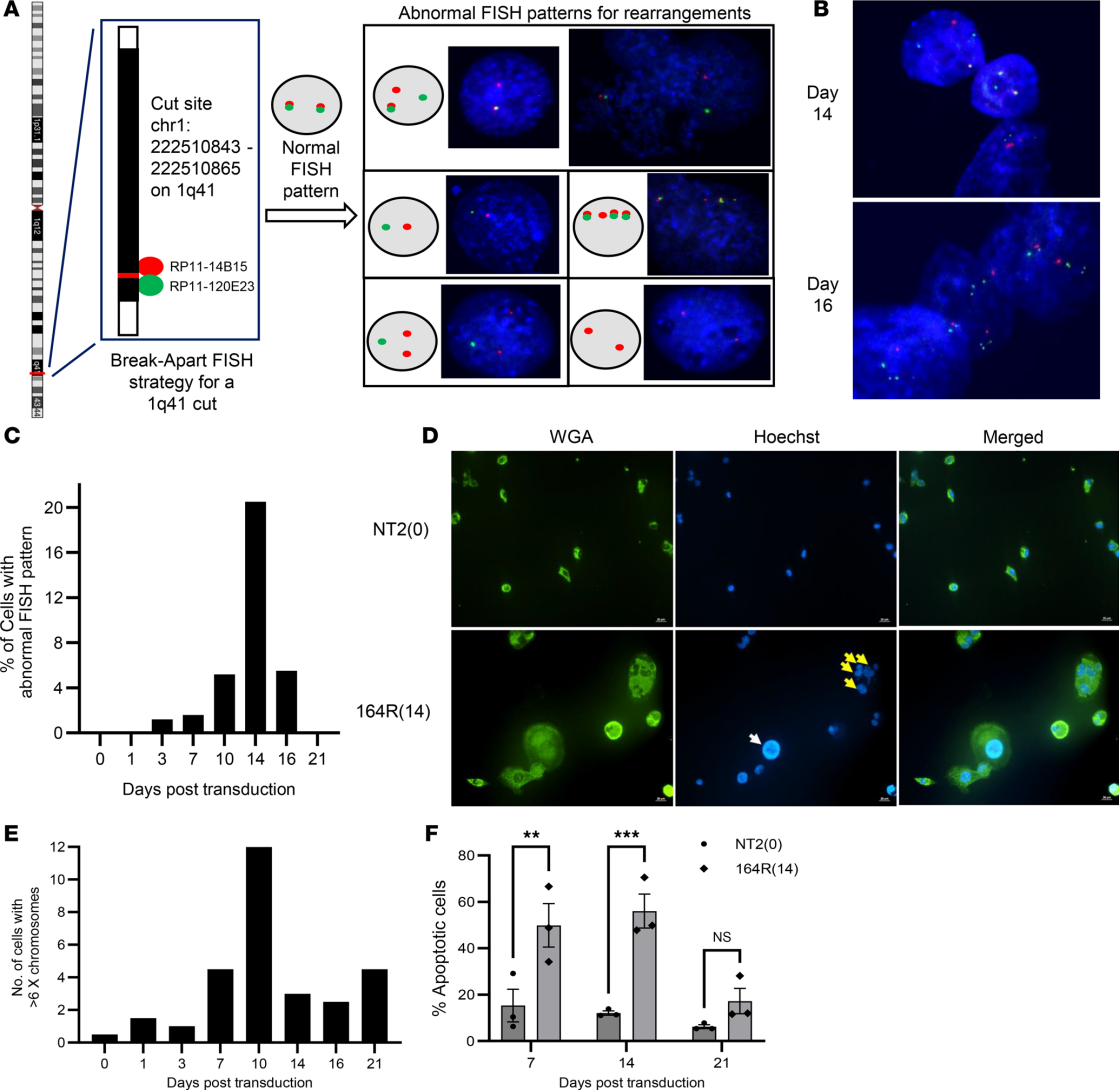
Peak polyploidy and chromosomal rearrangements in multitarget sgRNA–transduced cells. (**A**–**C**) TS0111 Cas9-expressing cells were transduced with 164R(14) sgRNA and subjected to break-apart FISH assays. (**A**) Break-apart FISH strategy at the 1q41 cut site. Abnormal FISH patterns were shown using cells collected at early time points. DNA was stained with DAPI. *N* = 1. (**B**) Complex rearrangements were observed in cells 14 and 16 days after transduction. *N* = 1. (**C**) Percentage of cells with rearrangements at 1q41 detected by break-apart FISH assay over time. (**D**) Shown are Panc10.05 cells transduced with NT2 or 164R(14) and stained with wheat germ agglutinin (WGA; green) and Hoechst 33342 (blue) 14 days after transduction. White arrow: large nucleus; yellow arrows: multiple nuclei in a cell. *N* = 3. (**E**) Number of TS0111-transduced cells with >6 X chromosomes over time using XY FISH. (**F**) Apoptosis analysis of Panc10.05 cells after treatment with 164R(14) or NT2 using annexin V flow cytometry assay. Šidák’s multiple comparisons test, day 7: **P = 0.005, day 14: ***P = 0.0008, and day 21: *P* = 0.53. *N* = 3; mean ± SEM.

To identify chromosomal changes after multiple CRISPR/Cas9 scissions, we conducted cytogenetic analyses on cells harvested from 0 to 21 days after transduction of a multitarget sgRNA at 3- to 4-day intervals using a detailed chromosome breakage assay. The TS0111 Cas9-expressing cell line was selected for its simpler karyotype compared with Panc10.05 (Supplemental Figure 6A). We chose the 164R(14) sgRNA because of its pronounced growth inhibition in previous assays. On the first day after transduction with 164R(14), multiple chromosome and chromatid breaks, along with radial formations, were detected (Figure 4A). Additional chromosomal aberrations accumulated over time, including ring, dicentric, and tricentric chromosomes; telomere-telomere associations; chromosome pulverizations; and endomitosis (Figure 4, A and B and Supplemental Figure 6B). Most aberrations peaked at day 14 and decreased by day 21, while chromosome/chromatid breaks remained stable throughout the experiment, indicating peak CIN during ongoing breakage events (Figure 4, A and B). We analyzed breakpoints of dicentric, tricentric, and ring chromosomes to determine their occurrence in CRISPR/Cas9-targeted versus nontargeted regions. Although SVs at targeted regions predominated at early time points, the majority of SVs occurred at nontargeted regions and peaked at day 14 (Figure 4C), consistent with ongoing chromosomal rearrangements. Notably, while most targeted regions were at telomeric regions, 61.5% of SVs at nontargeted regions were also located at telomeric sites (Supplemental Figure 6C). To investigate whether SVs at targeted regions were direct outcomes of CRISPR/Cas9 targeting, we performed a break-apart fluorescence in situ hybridization (FISH) assay at one of the 164R(14) sgRNA target sites (Figure 5, A–C). Simple rearrangements were observed at early time points (Figure 5A), whereas more complex rearrangements appeared later (Figure 5B). The number of cells with abnormal FISH patterns increased over time and peaked at day 14 (Figure 5C), demonstrating ongoing chromosomal rearrangements from the initial CRISPR/Cas9 scissions.

TS0111 cells also exhibited polyploidy in response to the 164R(14) sgRNA, characterized by extremely large nuclei or multinucleated giant cells, with >100 chromosomes per cell detected by cytogenetic analyses (Figure 5D and Supplemental Figure 6, D and E). An X/Y FISH assay to count cells with multiple (>6) X chromosomes revealed that polyploidy peaked at day 10 and decreased by day 21 (Figure 5E). To assess if apoptosis was involved in the cell death mechanism, we analyzed apoptosis markers in cells transduced with 164R(14). The proportion of apoptotic cells stained with annexin V increased on days 7 and 14, compared with cells transduced with a nontargeting sgRNA, but decreased by day 21 (Figure 5F). We consistently encountered difficulties harvesting enough viable cells for flow cytometry analyses on day 21. However, a TUNEL assay indicated increased late-stage apoptosis on day 21 (Supplemental Figure 6F).

**Figure 6:**
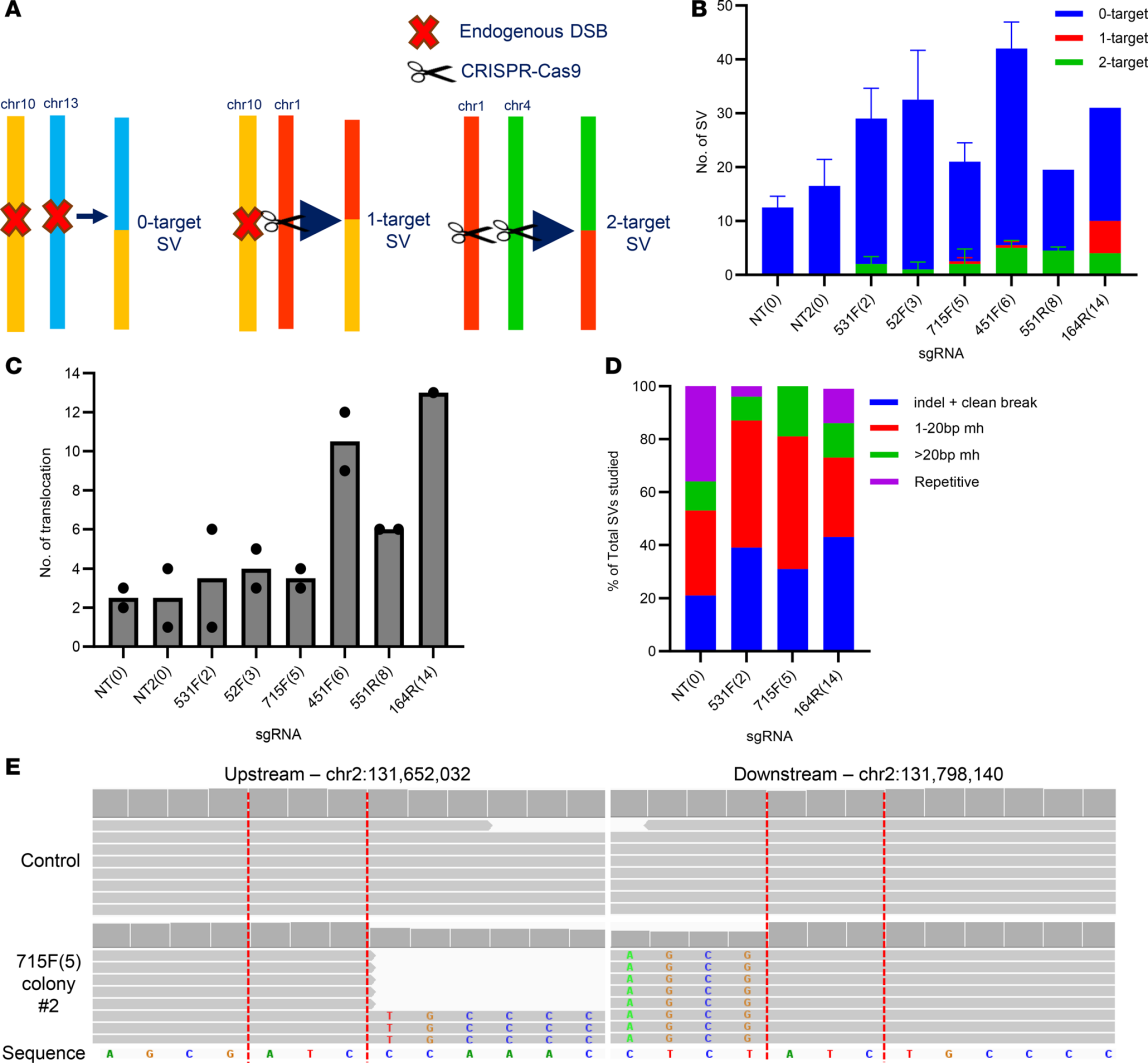
The majority of SVs are not directly produced from the initial CRISPR/Cas9-induced DSBs. (**A**) SVs were categorized by whether the breakpoints resulted from noninduced DSBs (0-target SV), from 1 site that was CRISPR/Cas9 targeted (1-target SV), or from both sites being targeted (2-target SV). (**B**) Quantification of SVs through WGS analyses of Panc10.05 surviving/resistant colonies after treatment with multitarget sgRNAs. *N* = 2 except for 164R(14) (*N* = 1); mean ± SEM. (**C**) Number of translocations detected in each Panc10.05 surviving colony. *N* = 2 except for 164R(14) (*N* = 1); mean ± SEM. (**D**) Sequences at breakpoint junctions were analyzed to identify indels and microhomology sequences (mh). Shown are the percentages of breakpoint types in each surviving colony. *N* = 2 except for 164R(14) (*N* = 1); mean only. (**E**) Example of a 0-target deletion from a 715F(5) sgRNA–resistant colony. The red dotted lines indicate the 3 bp homology region on both upstream and downstream sequences.

We also analyzed surviving colonies from our clonogenicity assays using WGS to identify, categorize, and quantify SVs absent in the parental control. This approach enabled us to examine DSB repair effects at both targeted and nontargeted regions with high resolution. Using the SV detection software manta (42), we identified SVs in surviving/resistant colonies previously transduced with multitarget sgRNAs, followed by visual inspection of all identified SVs using IGV (Figure 6). Our data revealed that while CRISPR/Cas9-induced SVs (1- and 2-target SVs) increased with the number of sgRNA target sites, the majority (87%) were noninduced DSBs (0-target SVs), which peaked in 451F(6) sgRNA–resistant colonies and subsequently decreased in 551R(8)- and 164R(14)-resistant colonies (Figure 6, A and B). Notably, translocations increased with the number of sgRNA target sites (Figure 6C). We analyzed sequences at breakpoint junctions for indels and microhomology (Figure 6, D and E), which could indicate involvement of nonhomologous end joining (NHEJ), microhomology-mediated end joining (MMEJ), and single-strand annealing (43, 44). We found that cells transduced with multitarget sgRNAs exhibited a higher proportion of breakpoints involving 1–20 bp microhomologies compared with the NT control (Figure 6D and Supplemental Table 5), suggesting the involvement of MMEJ in DSB repair. Overall, both cytogenetics and WGS analyses reveal that CRISPR/Cas9 scissions result in continuous chromosomal rearrangements that reach a tolerable limit, indicating that CRISPR/Cas9-induced extreme CIN is detrimental to cell survival.

### CRISPR/Cas9-induced DSBs cause higher cytotoxicity than irradiation.

**Figure 7:**
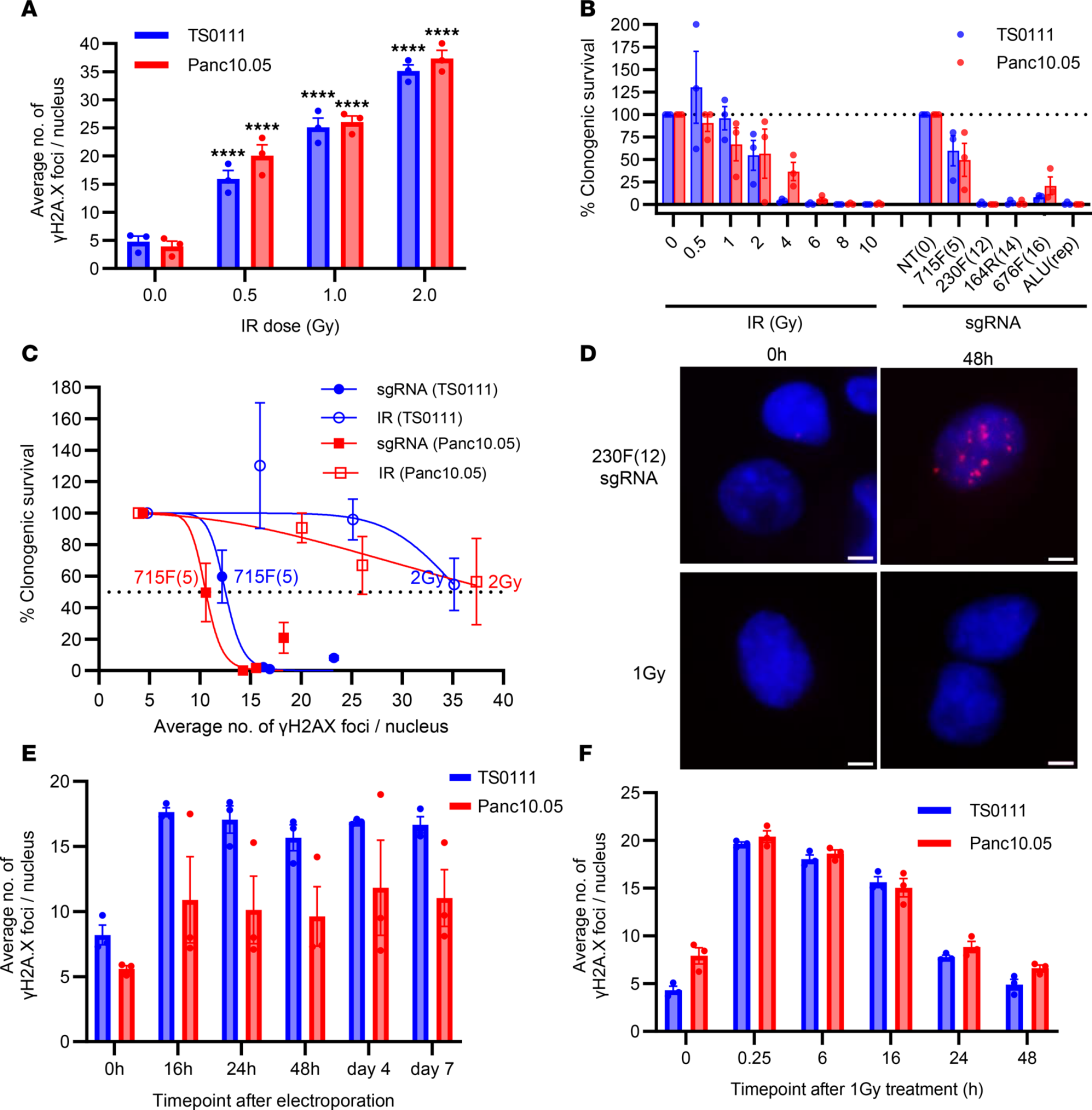
CRISPR/Cas9-induced DSBs are more cytotoxic than IR-induced DSBs. (**A**) Number of γH2A.X foci as a function of IR dose. >100 nuclei were analyzed for each condition. Dunnett’s test between 0 Gy and each dose; ****P < 0.0001. *N* = 3; mean ± SEM. (**B**) Clonogenic survival as a function of IR dose or number of sgRNA target sites after 21 days. *N* = 3; mean ± SEM, normalized to 0 Gy or NT. (**C**) Clonogenic survival with increased number of γH2A.X foci detected in IR- and sgRNA-treated cells. *N* = 3; mean ± SEM, nonlinear regressions were shown. (**D**) Representative merged images of γH2A.X (red) and DNA (DAPI, blue) staining in TS0111 cells treated with CRISPR/Cas9 RNP containing 230F(12) sgRNA or 1 Gy at 0- and 48-hour time points. Images at 40× original magnification; scale bar is 5 μm. *N* = 3. (**E** and **F**) Number of γH2A.X foci over time after (**E**) electroporating in CRISPR/Cas9 RNP containing 230F(12) sgRNA or (**F**) IR with 1 Gy. >100 nuclei were analyzed for each condition. *N* = 3; mean ± SEM.

This CRISPR/Cas9-induced CIN resembles observations from irradiation (IR) studies, where delayed CIN and cell death manifest over several generations after IR exposure (45–47). We compared the cytotoxicity of CRISPR/Cas9-induced DSBs with IR-induced DSBs to investigate their differential effects on cancer cell growth. We first quantified DSBs induced by irradiating Panc10.05 and TS0111 with 0–10 Gy and performing γH2A.X staining, showing a clear dose-response induction of DSBs (Figure 7A and Supplemental Figure 7, A and B). Cells treated with 4 Gy and higher exhibited an excessive number of foci that were uncountable (data not shown).

To compare the effects of IR-induced versus CRISPR/Cas9-induced DSBs on cell survival, we assessed clonogenic inhibition in Panc10.05 and TS0111 cells following either IR or multitarget sgRNA transduction. Clonogenic inhibition increased with both IR dose and the number of sgRNA target sites 21 days posttreatment (Figure 7B). Notably, the 715F(5) sgRNA induced similar clonogenic inhibition to 2 Gy IR (~55% and ~56%, respectively). More than 90% clonogenic inhibition was observed after 4 Gy in TS0111 and 6 Gy in Panc10.05, while more than 98% clonogenic inhibition was seen in 230F(12) sgRNA–treated cells from both lines. Cell viability assays supported these clonogenicity findings (Supplemental Figure 7, C and D). We also tracked cell survival in IR-treated cells every 3–4 days over 21 days, confirming that increased IR doses decreased cell numbers, and TS0111 demonstrated higher sensitivity to IR than Panc10.05 (Supplemental Figure 7, E and F).

We plotted clonogenic survival against γH2A.X foci and found that a much smaller number of DSBs induced by CRISPR/Cas9 compared with IR yielded similar levels of clonogenic inhibition (Figure 7C). For instance, while both 715F(5) sgRNA and 2 Gy IR resulted in comparable reductions in clonogenic survival, the γH2A.X foci in 715F(5)-treated cells (~11 foci) were markedly fewer than those in 2 Gy–treated cells (~36 foci). Radiation doses of 4–10 Gy were highly cytotoxic to both cell lines, leading to nearly complete loss of survival, accompanied by an excessive number of foci that were uncountable. Interestingly, similar growth inhibition was observed in 230F(12)- and 164R(14)-treated cells, which had substantially fewer γH2A.X foci than those induced by equitoxic radiation doses. Our data suggest that CRISPR/Cas9-induced DSBs are considerably more cytotoxic than a comparable number of IR-induced DSBs.

Given that the cytotoxicity from CRISPR/Cas9-induced DSBs arises from ongoing chromosomal rearrangements, we hypothesized that the lower cytotoxicity of IR-induced DSBs could stem from transient DSB formation followed by immediate repair, as opposed to persistent DSBs from CRISPR/Cas9 scissions. We electroporated CRISPR/Cas9 RNP complexes containing 230F(12) sgRNAs into both PC cell lines, quantified their γH2A.X foci over time, and compared them with cells treated with 1 Gy (Figure 7, D–F, and Supplemental Figure 8). Since electroporated cells were not fully attached prior to the 16-hour (h) time point, data before 16 h were excluded to avoid potential confounding effects. Our data indicated that 230F(12) sgRNA treatment led to persistent γH2A.X foci formation up to our final time point (day 7), with foci peaking and plateauing starting at 16 h (Figure 7, D and E, and Supplemental Figure 8A). High variability was observed in Panc10.05, likely due to the presence of cells that did not receive the CRISPR/Cas9 RNP complex and survived. In contrast, γH2A.X expression in irradiated cells peaked at 15 minutes (mins) and decreased over time, returning to baseline levels at 48 h (Figure 7, D and F, and Supplemental Figure 8B), indicating completion of DSB repair within that time frame. To confirm that electroporation alone did not increase γH2A.X expression, we introduced an NT sgRNA and counted foci at 48 h, revealing an average of 5.9 foci when counting 100 cells, consistent with our baseline data. Thus, our findings suggest that while IR-induced DSBs are rapidly repaired, CRISPR/Cas9-induced DSBs persist for an extended period.

### Cells resistant to one sgRNA retain sensitivity to other sgRNAs and do not exacerbate disease.

**Figure 8:**
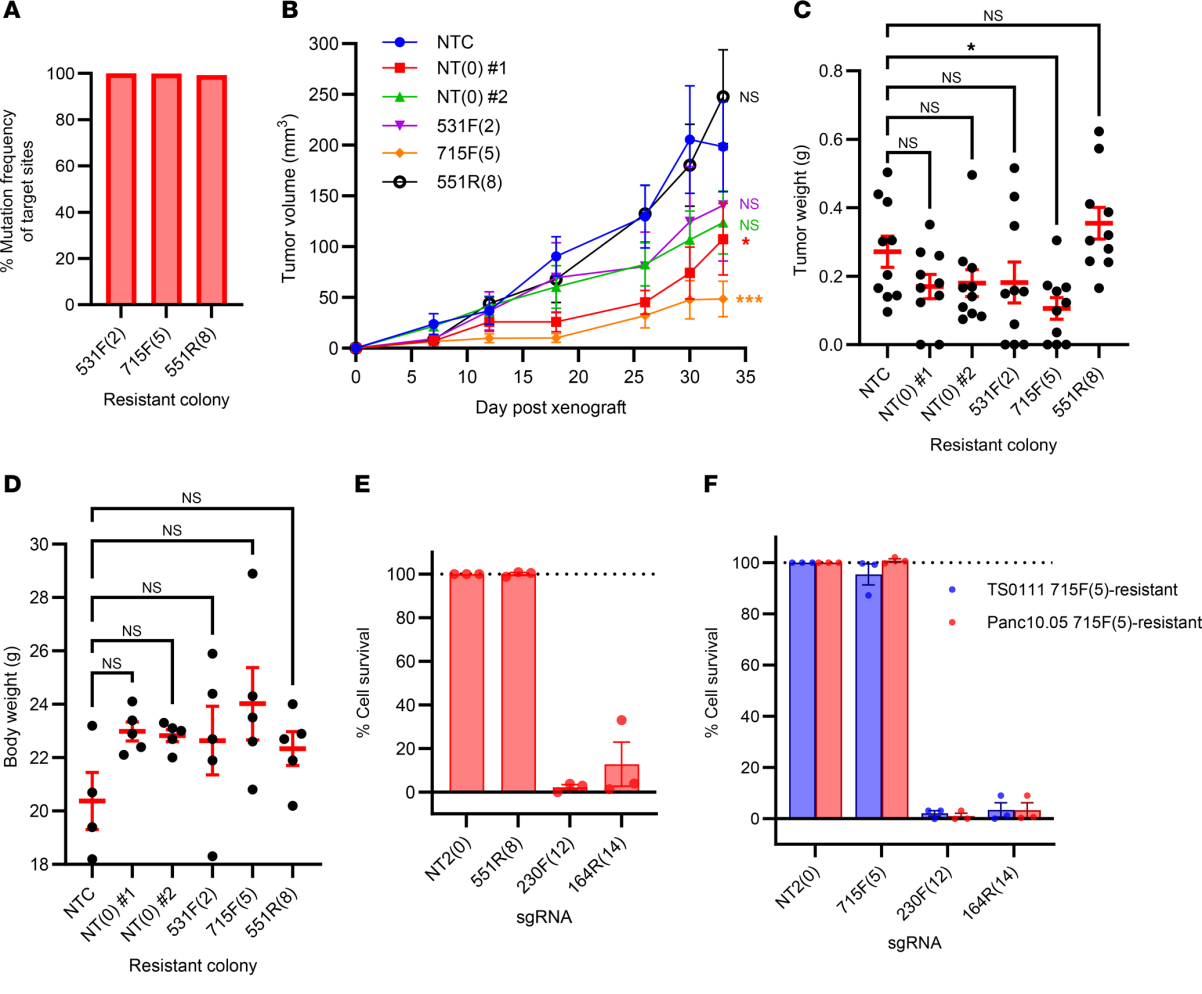
Cells resistant to one sgRNA are susceptible to other sgRNAs. (**A**) Mutation frequency of sgRNA target sites in each CRISPR/Cas9 surviving colony used for xenograft experiment. (**B**–**D**) Tumor growth experiment of CRISPR/Cas9 surviving colonies in subcutaneous xenograft models. In addition to a nontransduced cell line (NTC), surviving colonies from clonogenicity experiment (Figure 1A) transduced with NT sgRNA (NT colony #1 and NT colony #2) and multitarget sgRNAs 531F(2), 715F(5), and 551R(8) were injected into nude mice for tumor growth. (**B**) Tumor volume measurements postxenograft. Dunnett’s test between NTC and NT #1: *P* = 0.050, NT #2: *P* = 0.145, 531F(2): *P* = 0.349, 715F(5): *P* = 0.0002, 551R(8): *P* = 0.498 on week 5. *N* = 10 (*N* = 8 for NTC on day 30 and 33 due to early death); mean ± SEM. (**C**) Tumor weight measurements. Dunnett’s test between NTC and NT #1: *P* = 0.341, NT #2: *P* = 0.437, 531F(2): *P* = 0.457, 715F(5): *P* = 0.041, and 551R(8): *P* = 0.531. *N* = 10; mean ± SEM. (**D**) Body weight of mice 5 weeks postxenograft. Dunn-Šidák test between NTC and the other treatment groups showed no significant differences. *N* = 5 except for NTC (*N* = 4 due to early death); mean ± SEM. (**E**) Cell survival of Panc10.05 551R(8)–resistant colony that was retransduced with nontargeting sgRNA (NT2) or multitargeting sgRNAs — 551R(8), 230F(12), and 164R(14) — as detected by alamar blue cell viability assay and normalized to NT2. *N* = 3; mean ± SEM. (**F**) Cell survival of TS0111- and Panc10.05 715F(5)–resistant colony that was retransduced with nontargeting sgRNA (NT2) or multitargeting sgRNAs — 715F(5), 230F(12), and 164R(14) — as detected by alamar blue cell viability assay and normalized to NT2. *N* = 3; mean ± SEM.

To investigate whether surviving/resistant colonies from the clonogenicity experiment (Figure 1A) enhanced tumor growth compared with nontransduced cells, we first assessed mutations at the multitarget sgRNA target sites in each colony using deep NGS, confirming their resistance against CRISPR/Cas9-induced DSBs (Figure 8A). We found that the overall mutation frequency of all target sites in each colony exceeded 99%, indicating that these colonies had their target sites mutated during the initial sgRNA transduction. We then injected both parental and resistant cells subcutaneously into nude mice and monitored tumor growth over 33 days (Figure 8B). We observed decreased tumor growth in 1 of the 2 NT controls (NT #1) and in 715F(5)-resistant tumors [715F(5)], while there were no significant differences in growth for other resistant tumors compared with the parental/nontransduced control (NTC). We harvested tumors 33 days postxenograft or postmortem (Supplemental Figure 9A) to measure their weights, and we found no significant differences between resistant tumors and NTCs, except for the 715F(5)-resistant tumors, which displayed reduced weight (Figure 8C). No differences in body weight were noted (Figure 8D). Thus, our results indicate that DSB-resistant cancer cells induced by CRISPR/Cas9 do not grow significantly faster than the nontransduced or NT-transduced cells in subcutaneous xenograft mouse models.

Finally, we hypothesized that cells surviving the initial CRISPR/Cas9-induced DSBs could be eliminated by a subsequent round of CRISPR/Cas9-induced DSBs. Using surviving colonies from our clonogenicity assay, with a subset of their target sites validated for mutations via NGS (Supplemental Figure 9B), we retransduced these colonies with NT sgRNA (NT#2), multitarget sgRNA already present in the colonies [e.g., 715F(5)-resistant colonies were treated with 715F(5) sgRNA], 230F(12) sgRNA, and 164R(14) sgRNA. After 21 days, we observed no growth inhibition in cells treated with the NT control or those retransduced with their original sgRNAs, but we found >96% inhibition with the 230F(12) sgRNA and >87% with the 164R(14) sgRNA (Figure 8, E and F, and Supplemental Figure 9C). We collected double-resistant colonies (i.e., cells that survived both the original and secondary transductions) and performed NGS on their target sites. In 230F(12)-resistant colonies, the 230F(12) target sites were not mutated (Supplemental Figure 9D), suggesting that CRISPR/Cas9 activity from the secondary transduction was absent in these double-resistant colonies. We detected ~38% mutation frequency in colonies transduced with the 164R(14) sgRNA (Supplemental Figure 9D), indicating that most surviving cells lacked CRISPR/Cas9 activity from the secondary transduction.

## Discussion

In this study, we show that simultaneous induction of multiple CRISPR/Cas9-induced DSBs overwhelms the DNA repair machinery, leading to extreme CIN and cell death — a phenomenon we term “chromosome catastrophe.” This is elucidated especially in our rare surviving colonies, which reveal that most SVs arise from this catastrophic process rather than direct CRISPR/Cas9 targeting.

Additionally, CRISPR/Cas9-induced DSBs are markedly more cytotoxic than comparable doses of IR, exhibiting persistent DSBs in sgRNA-treated cells versus transient DSBs in IR-treated cells. This is likely due to repeated DNA cleavage cycles at CRISPR/Cas9 target sites until NHEJ-mediated errors alter the sgRNA recognition sequence, stopping further cutting (48). Such repeated cleavages have been noted in endonuclease-based systems unless the endonuclease activity is turned off (49, 50). In contrast, IR induces DSBs in a single pulse followed by immediate repair. The γH2A.X foci count in multitarget sgRNA–transduced cells may underestimate DSBs, as WGS often detects more DSBs; e.g. γH2A.X staining revealed 12 foci in TS0111 715F(5)-treated cells, while WGS detected 16 DSBs. Even accounting for background DSB formation (~4 foci) and WGS-detected DSBs from CRISPR/Cas9, the total remains lower than that from IR. Consequently, targeting 6–8 genomic sites with CRISPR/Cas9 results in similar cancer cell kill as 2 Gy IR, underscoring its therapeutic potential to induce tumor cell death similar to clinically relevant doses of radiation (51). Combining CRISPR/Cas9-induced DSBs with current chemotherapies that induce CIN, such as paclitaxel, or other agents that induce CIN selectively, such as KIF18A inhibitors, may further enhance cancer cell elimination (52–55).

Our sgRNA design strategy has led to most multitarget sgRNA sites being located near telomeres, which may contribute to the observed cell death. Cells treated with multitarget sgRNA exhibited CIN features characteristic of telomere crisis, such as extensive chromosomal rearrangements and endoreduplication, leading to a high cell death rate (56, 57). Umbreit et al. demonstrated that targeting the subtelomere of chromosome 4 in telomerase-immortalized RPE-1 cells with CRISPR/Cas9 generated chromosome bridges, supporting the hypothesis that targeting telomeric regions induces CIN (19). We observed that microhomologies were involved in most breakpoints in multitarget sgRNA–transduced colonies, aligning with literature suggesting that MMEJ plays a significant role in CRISPR/Cas9-induced DSB repair (58–60). A potential avenue for further research could involve comparing the cytotoxicity of near-telomeric versus near-centromeric targeting and exploring whether cotreatment with an MMEJ inhibitor could enhance cytotoxicity.

Polyploidization, while generally linked to therapeutic resistance (61–63), likely amplifies CIN in multitarget sgRNA–treated cells, leading to lethality rather than tumorigenicity. This could explain the absence of surviving colonies from certain treatments after 3 months. We observed few SVs at CRISPR/Cas9 on-target sites in surviving colonies, though CRISPR/Cas9-induced rearrangements were present in our break-apart FISH assay, suggesting these colonies may exhibit lower CIN compared with nonsurvivors. Future research should explore whether these colonies arise from polyploid progenies and compare their CIN rates to the bulk population.

Combining cytogenetic analysis with WGS provided complementary insights: (a) cytogenetic analysis visualizes chromosomal breakpoints directly, potentially offering a more accurate reflection than SV-calling software, which can be affected by mapping errors in repetitive regions (64); (b) WGS identifies the nucleotide sequences of breakpoints and assesses potential off-target activity at higher resolution compared to cytogenetics; (c) cytogenetics reveals chromosomal aberrations contributing to CIN, while WGS relies on parameters to accurately identify structural abnormalities; and (d) WGS detects smaller variations like indels and SNVs often missed by cytogenetics because of resolution limits. Thus, despite advancements in SV callers, cytogenetics remains vital for assessing genome-wide rearrangements at the single-cell level.

While we measured the copy number of individual genomic sites using WGS in the Cas9-derived line before CRISPR/Cas9 treatment, we recognize that surviving clones might exhibit unique aneuploid distributions because of genomic instability and subclonality. Our WGS analyses, conducted on 2 surviving clones per treatment, aimed to mitigate background variability. We acknowledge that subclones within malignant tumors can differ from the bulk cancer. In our rapid autopsy study of 5 patients, we identified truncal loss-of-homozygosity regions consistent across metastases, despite overall genomic instability (65).

We show that cells surviving CRISPR/Cas9 scission do not exhibit enhanced tumor growth, likely because scissions did not occur in tumor suppressor genes. Importantly, these survivors remain sensitive to further targeting, indicating resistance does not develop easily. This suggests CRISPR/Cas9 could serve as a targeted therapeutic that leverages multiple DSBs rather than modulating specific oncogenic pathways.

Successful delivery is crucial for targeting cancer in vivo. Viral delivery, commonly used in gene therapy, faces many limitations and risks, including immunogenicity, packaging capacity, and regulatory concerns (66–68). Nanoparticles, particularly lipid nanoparticles (LNPs), encapsulating Cas9 mRNA and therapeutic sgRNAs, have shown promising efficacy and safety in early-phase clinical trials (69–71). Although our safety data suggest well-tolerated delivery to normal cells, additional strategies may be needed to ensure preferential delivery to cancer cells (72, 73). Virus-like particles and exosome-based systems are emerging as gene therapy delivery platforms (74, 75), and more clinical data will be needed to assess their therapeutic potentials. As LNP delivery to the liver is well established and liver is a common site of metastases for various cancers (e.g., breast, colorectal, pancreatic, lung, melanoma) (76), our initial clinical trials could focus on treating liver metastases.

In summary, we demonstrate that a small number of CRISPR/Cas9-induced DSBs in noncoding regions can cause PC cell death, with a cytotoxicity more potent than IR because of accumulated CIN events leading to chromosome catastrophe. We also show that by using sgRNAs that are specific to a patient’s cancer, we could achieve tumor growth inhibition. Given the urgent need for improved therapies for PC, this study highlights the potential of CRISPR/Cas9 as a distinct and selective cell killing strategy against PC.

## Methods

### Sex as a biological variable.

All mouse experiments were double-blind and used only female mice for practical reasons (e.g., less fighting).

### Mice.

Protocols for generating the Cas9-expressing cell line for xenograft and the xenograft-adapted parental line can be found in the Supplemental Methods. In the subcutaneous xenograft experiment using pretreated cells, Cas9-expressing Panc10.05 cells were transduced with lentivirus containing sgRNA-expressing plasmids at MOI 10. Following 7 days of puromycin selection, 5 × 10^5^ cells per tumor in 100 μL PBS were subcutaneously injected into the flanks of randomized, 12-week-old, female, nude, athymic mice (Envigo). Each mouse received 2 tumors, and body weight and tumor volume were measured weekly by an investigator under a blinded protocol. Mice were monitored for adverse effects, and 6 weeks postxenograft, tumors were surgically extracted and weighed by 2 investigators under a blinded protocol.

For the hemispleen injection model assessing PC liver metastasis, 8-week-old, female, nude mice were randomized and underwent hemisplenectomy following the pretreatment of cells. After a week of puromycin selection, 1 × 10^6^ cells were injected into half of the spleen to seed the liver. Mice were anesthetized using isoflurane (2 L/min O_2_, 2% isoflurane), and sterile surgical techniques were employed. Toe pinch was administered to ensure mice were at optimal anesthetic depth. Scissors and forceps were used to make a 1 cm linear incision in the dermis and peritoneal cavity and expose the spleen. Two titanium ligating clips were placed near the center of the spleen. Using scissors, the spleen was cut in half between the 2 ligating clips. The anterior half was placed back into the peritoneal cavity. Using a 1 mL TB syringe with a 25-gauge needle, 50 μL of PBS was aspirated followed by 100 μL of tumor cell suspension in DMEM tissue culture media. The final volume of 150 μL was injected into the posterior half of the spleen slowly. After several seconds, 2 titanium ligating clips were used to clamp the vessels descending from the posterior half of the spleen before removing the posterior half. A 4-0 absorbable silk suture was used to create a continuous double stitch in the mucosa layer followed by 2 discrete sutures to close the skin incision. Finally, a surgical clamp was used to secure the incision further. Following surgery, mice were placed on a warming table and monitored for 30 minutes. Mice were assessed daily for discomfort. Livers were harvested 30 days after surgery, and the left lobes were fixed in 10% formalin at room temperature for 48 hours and paraffin-embedded. We collected 5 μm tissue sections at 250 μm levels between each section, yielding approximately 10 slides per liver. Tissue sections were H&E-stained and evaluated by a liver pathologist.

The protocol for the Dox-iCas9 mouse experiment can be found in Supplemental Methods. For the hydrodynamic injection experiment, the establishment of a hemispleen injection model was the same as previously described. The hydrodynamic injection protocol and luciferase protein detection are described in Supplemental Methods.

In the surviving colonies experiment, cells were maintained under blasticidin and puromycin selection for Cas9 and sgRNA expression before being injected into 6-week-old, female, nude mice (Envigo). Mice were randomized and received 5 × 10^6^ cells per tumor in 50 μL Matrigel (Corning) subcutaneously in both flanks. Body weight and tumor volume were measured weekly by a blinded investigator, and tumors were surgically extracted for weighing 5 weeks postxenograft by 2 blinded investigators.

### Clonogenicity and cell viability assays.

For multitarget sgRNA transduction, cells were transduced with lentivirus carrying sgRNA-expressing plasmids at MOI 10 in media containing 10 μg/mL polybrene. Cell culture conditions are detailed in Supplemental Methods. After 20 h of incubation, cells were washed once with 1× PBS and returned in normal media. The next day, cells were diluted 1:1,000 and plated in 96-well plates for clonogenic survival under 1 μg/mL puromycin or 200 μg/mL hygromycin selection. For electroporation of CRISPR/Cas9 RNP complex (protocol in Supplemental Methods), electroporated cells were plated evenly in 96-well plates for clonogenic growth. For normal cell line transduction, MOI was increased to 50, with 500 cells/well in 96-well plates. Negative controls included equivalent doses of NT and NT2 sgRNAs, while positive controls consisted of AGGn, L1.4_209F, and ALU_112a sgRNAs. For radiation, cells were treated with 0–10 Gy (CIXD X-RAY irradiator, xstrahl) and plated at 1:1,000 and 1:10,000 dilutions on the same day. For all experiments, at the specified endpoints described in Results or figure legends, colonies were counted using phase microscopy, and alamar blue Cell Viability Reagent (Thermo Fisher Scientific) was added per the manufacturer’s instructions. Fluorescence readings were performed on a BMG POLARstar Optima microplate reader, setting excitation at 544 nm and emission at 590 nm, with a gain of 1,000 and required value of 90%.

*γH2A.X staining and imaging*. Cells were seeded at a density of 2 × 10^5^ cells/well of a 6-well plate with coverslips. For transduction of multitarget sgRNAs, cells were transduced and washed as described above. At 48 h posttransduction or at predetermined time points after electroporation, cells were fixed. For the radiation experiment, cells were treated with the indicated doses of radiation (CIXD X-RAY irradiator) and fixed at predetermined time points after radiation. For both experiments, cells were fixed with 4% paraformaldehyde for 20 mins at room temperature. Cells were then washed twice with 1× PBS for 5 mins each, blocked, and permeabilized with 5% BSA/0.5% Triton X-100 in PBS for 30 mins (mins) at room temperature, followed by an overnight incubation with anti–phospho-histone H2A.X (Ser139) antibody (05-636, Sigma-Aldrich) at 1:1,000 dilution. The next day, cells were washed thrice with PBS for 5 mins each and subsequently incubated with secondary antibody conjugated with Alexa Fluor 594 (A-11032, Thermo Fisher Scientific) for 1 h at room temperature. Cells were again washed thrice with PBS and then counterstained with DAPI-containing mounting medium (H-1800, Vector Labs). Stained cells were imaged using a fluorescence microscope (ZEISS AxioImager Z1) with a 40× objective. γH2A.X foci were counted manually under the microscope with a minimum of 100 nuclei for each sample.

### WGS of surviving colonies.

Genomic DNA was extracted from surviving colonies of clonogenicity assay using QIAamp UCP DNA Micro Kit (QIAGEN) according to the manufacturer’s protocol. SKCCC Experimental and Computational Genomics Core sent the samples to New York Genome Center for WGS with an Illumina HiSeq 2000 using the TruSeq DNA prep kit. Sequencing was carried out so as to obtain 30× coverage from 2 × 100 bp paired-end reads. FASTQ files were aligned to both hg19 and hg38 using bwa v0.7.7 (77) to create BAM files. The default parameters were used. Picard-tools1.119 was used to add read groups as well as remove duplicate reads. GATK v3.6.0 (78) base call recalibration steps were used to create a final alignment file.

### sgRNA tag survival assay.

Cells were transduced with a lentivirus pool containing all the sgRNAs in Supplemental Table 1 at MOI 0.1 into media containing 10 μg/mL polybrene. After 24 h, approximately 1 million cells were collected for day 1 time point, and the remaining cells were cultured for another day, then subjected to 1 μg/mL puromycin selection. Cells were collected on days 1, 7, 14, and 21 posttransduction, and gDNA extractions were performed using QIAamp UCP DNA Micro Kit (QIAGEN) by following the manufacturer’s protocol. sgRNA library was prepared by amplifying the sgRNA target region from gDNAs using NGS primers provided by Joung et al. (79), based on the protocol outlined in the paper, and sent for NGS (primers in Supplemental Methods). Read counts of each sgRNA were extracted from FASTQ files using the script Joung et al. (79) provided and were put through the MAGeCK (80) pipeline to obtain sgRNA fold-change.

### Chromosome breakage assay.

TS0111-Cas9-EGFP cells plated at 5 × 10^5^/mL were treated with 164R(14) sgRNA and harvested at 0, 1, 3, 7, 10, 14, 16, and 21 days. Colcemid (0.01 μg/mL) was added 20 h before harvesting. Cells were then exposed to 0.075 M KCl hypotonic solution for 30 mins, fixed in 3:1 methanol/acetic acid, and stained with Leishman’s for 3 mins. For each treatment, 100 consecutive analyzable metaphases were analyzed for induction of chromosome abnormalities including chromosome/chromatid breaks and exchanges.

### Statistics.

The appropriate statistical tests were performed in GraphPad Prism (Version 9.2.0). The statistical models used are stated in Results and figure legends. Statistical significance was set at *P* < 0.05.

### Study approval.

Mouse experiments were approved by The Johns Hopkins University Animal Care and Use Committee (MO24M222). Cell line collections and uses from patient samples were approved by The Johns Hopkins Medicine Institutional Review Boards (NA_00074387). The need for written informed consent was waived by the IRB.

### Data availability.

The authors confirm that data supporting the findings of this study are available in the article and supplemental materials when possible. Values for all data points in graphs are reported in the Supporting Data Values file. Constructed plasmids have been deposited at Addgene, and specific sgRNA-expressing plasmids can be requested. With the exception of Panc10.05, which is an ATCC line, all other cell lines and their derivatives (including Panc10.05-derived lines) are available through Material Transfer Agreements. Sequencing data cannot be shared publicly due to IRB restrictions on deidentified data in line with research participant consent. Researchers can request more detailed data from the corresponding author through an approved collaboration arrangement.

## Author contributions

SSKT, AK, AB, NJR, MG, YSZ, and JRE conceived of and designed the study. SSKT, AK, AB, EHS, LM, and QZ conducted experiments. SZ, YS, AS, KB, HL, CFH, and RAA contributed to the acquisitions and analyses of data. CFH, RAA, NJR, RBS, MG, YSZ, and JRE provided resources and supervision. SSKT and JRE wrote the original draft of the manuscript. SSKT, AK, AB, EHS, LM, KB, RHH, CFH, RAA NJR, RBS, MG, YSZ, and JRE provided critical revisions to the manuscript. The authorship order among the co–first authors was determined to reflect their substantial contribution to the study, while acknowledging varying degrees of involvement in the collaborative work.

## Conflict of interest

SSKT, KB, NJR, JRE, and Johns Hopkins University have filed a patent application with the USPTO: US19/051,327. RAA received consulting fees from Bristol Myers Squibb, Gilead, Jazz Pharmaceuticals, GlaxoSmithKline, RAPT Therapeutics, Daiichi Sankyo, and AbbVie. Johns Hopkins University owns equity in Delfi Diagnostics. RBS is a founder of and holds equity in Delfi Diagnostics. RBS also serves as the head of data science.

## Funding support

This work is the result of NIH funding, in whole or in part, and is subject to the NIH Public Access Policy. Through acceptance of this federal funding, the NIH has been given a right to make the work publicly available in PubMed Central.

- National Institutes of Health grant R21CA164592 (JRE).
- National Institutes of Health grant P50CA62924 (SKCCC).
- National Cancer Institute CCSG P30CA006973 (SKCCC).
- The Sol Goldman Pancreatic Cancer Research Center (JRE).
- PanCan/AACR Innovation Award (JRE).
- The STRINGER Foundation (JRE).
- Casimir H. Zgonina Family Endowment for Pancreatic Cancer Research (JRE).
- Dennis Troper and Susan Wojcicki (JRE).
- Dick Knox/Cliff Minor Fund (JRE).
- Edward A. Goldsmith Pancreatic Cancer Research Fund (JRE).
- Elaine Crispen Sawyer Endowment (JRE).
- Elaine T. Koehler Pancreatic Cancer Research Fund (JRE).
- Eve Stancik Memorial Fund (JRE).
- George Rubis Endowment for Pancreatic Cancer Research (JRE).
- Hilda Buchfeller Yost Young Investigator’s Fund (JRE).
- James S. McFarland Endowment for Pancreatic Cancer Research (JRE).
- John J. Lussier Pancreatic Cancer Research Fund (JRE).
- Linda C. Talecki Gallbladder Cancer Research Fund (JRE).
- Mary M. Graf Memorial Endowment Fund for Gallbladder Cancer (JRE).
- Mary Lou Wootton Endowment (JRE).
- Professor J. Mayo Greenberg and Dr. Samuel L. Slovin Endowment (JRE).
- Rawlings Family Pancreatic Cancer Research Fund (JRE).

## Supplementary Material

Supplemental data

Supplemental data set 1

Supporting data values

## Figures and Tables

**Table 1:**
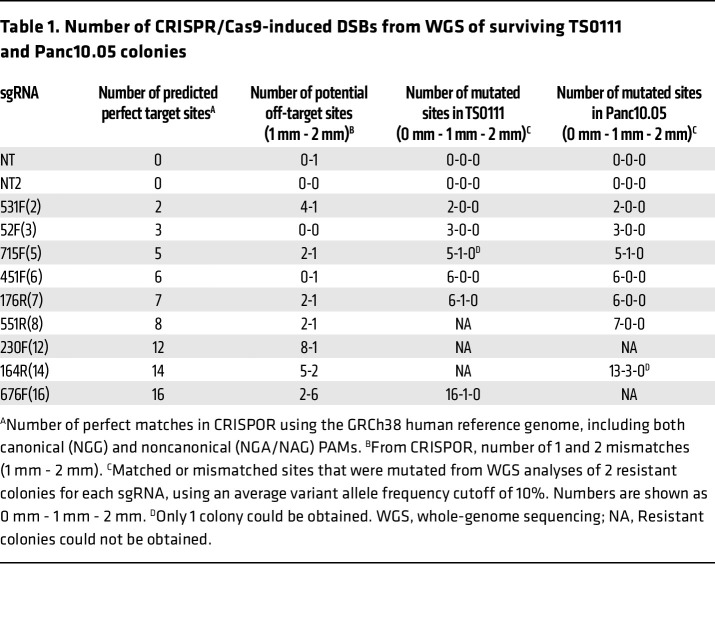
Number of CRISPR/Cas9-induced DSBs from WGS of surviving TS0111 and Panc10.05 colonies

## Version 1 05/15/2026

Electronic publication

## References

[1] Sansregret L (2018). Determinants and clinical implications of chromosomal instability in cancer. Nat Rev Clin Oncol.

[2] Sotillo R (2010). Mad2-induced chromosome instability leads to lung tumour relapse after oncogene withdrawal. Nature.

[3] Rowald K (2016). Negative selection and chromosome instability induced by Mad2 overexpression delay breast cancer but facilitate oncogene-independent outgrowth. Cell Rep.

[4] Birkbak NJ (2011). Paradoxical relationship between chromosomal instability and survival outcome in cancer. Cancer Res.

[5] Roylance R (2011). Relationship of extreme chromosomal instability with long-term survival in a retrospective analysis of primary breast cancer. Cancer Epidemiol Biomarkers Prev.

[6] Silk AD (2013). Chromosome missegregation rate predicts whether aneuploidy will promote or suppress tumors. Proc Natl Acad Sci U S A.

[7] Janssen A (2009). Elevating the frequency of chromosome mis-segregation as a strategy to kill tumor cells. Proc Natl Acad Sci U S A.

[8] Godek KM (2016). Chromosomal instability affects the tumorigenicity of glioblastoma tumor-initiating cells. Cancer Discov.

[9] Nickoloff JA (2020). Clustered DNA double-strand breaks: biological effects and relevance to cancer radiotherapy. Genes (Basel).

[10] Trenner A, Sartori AA (2019). Harnessing DNA double-strand break repair for cancer treatment. Front Oncol.

[11] Iliakis G (2019). Necessities in the processing of DNA double strand breaks and their effects on genomic instability and cancer. Cancers (Basel).

[12] McClintock B (1939). The behavior in successive nuclear divisions of a chromosome broken at meiosis. Proc Natl Acad Sci U S A.

[13] Lo AWI (2002). DNA amplification by breakage/fusion/bridge cycles initiated by spontaneous telomere loss in a human cancer cell line. Neoplasia.

[14] Li C (2023). Deciphering complex breakage-fusion-bridge genome rearrangements with Ambigram. Nat Commun.

[15] Schipler A (2016). Chromosome thripsis by DNA double strand break clusters causes enhanced cell lethality, chromosomal translocations and 53BP1-recruitment. Nucleic Acids Res.

[16] Aguirre AJ (2016). Genomic copy number dictates a gene-independent cell response to CRISPR/Cas9 targeting. Cancer Discov.

[17] Munoz DM (2016). CRISPR screens provide a comprehensive assessment of cancer vulnerabilities but generate false-positive hits for highly amplified genomic regions. Cancer Discov.

[18] Kosicki M (2018). Repair of double-strand breaks induced by CRISPR-Cas9 leads to large deletions and complex rearrangements. Nat Biotechnol.

[19] Umbreit NT (2020). Mechanisms generating cancer genome complexity from a single cell division error. Science.

[20] Kwon T (2022). Precision targeting tumor cells using cancer-specific InDel mutations with CRISPR-Cas9. Proc Natl Acad Sci U S A.

[21] Yang L (2015). Genome-wide inactivation of porcine endogenous retroviruses (PERVs). Science.

[22] Kuscu C (2017). CRISPR-STOP: gene silencing through base-editing-induced nonsense mutations. Nat Methods.

[23] Smith CJ (2020). Enabling large-scale genome editing at repetitive elements by reducing DNA nicking. Nucleic Acids Res.

[24] Niu D (2017). Inactivation of porcine endogenous retrovirus in pigs using CRISPR-Cas9. Science.

[26] Teh SSK (2024). CRISPR-Cas9 for selective targeting of somatic mutations in pancreatic cancers. NAR Cancer.

[27] Ihry RJ (2018). p53 inhibits CRISPR-Cas9 engineering in human pluripotent stem cells. Nat Med.

[28] Haapaniemi E (2018). CRISPR-Cas9 genome editing induces a p53-mediated DNA damage response. Nat Med.

[29] Enache OM (2020). Cas9 activates the p53 pathway and selects for p53-inactivating mutations. Nat Genet.

[30] https://seer.cancer.gov/statfacts/html/pancreas.html.

[31] Kleeff J (2016). Pancreatic cancer. Nat Rev Dis Primers.

[32] AACR Project GENIE Consortium (2017). AACR Project GENIE: powering precision medicine through an international consortium. Cancer Discov.

[33] Baskar R (2014). Biological response of cancer cells to radiation treatment. Front Mol Biosci.

[34] Bae S (2014). Cas-OFFinder: a fast and versatile algorithm that searches for potential off-target sites of Cas9 RNA-guided endonucleases. Bioinformatics.

[35] Sedelnikova OA, Bonner WM (2006). GammaH2AX in cancer cells: a potential biomarker for cancer diagnostics, prediction and recurrence. Cell Cycle.

[36] Yu T (2006). Endogenous expression of phosphorylated histone H2AX in tumors in relation to DNA double-strand breaks and genomic instability. DNA Repair (Amst).

[37] Concordet JP, Haeussler M (2018). CRISPOR: intuitive guide selection for CRISPR/Cas9 genome editing experiments and screens. Nucleic Acids Res.

[38] https://www.idtdna.com/site/order/designtool/index/CRISPR_CUSTOM.

[39] Graf R (2019). sgRNA sequence motifs blocking efficient CRISPR/Cas9-mediated gene editing. Cell Rep.

[40] Brinkman EK (2018). Kinetics and fidelity of the repair of Cas9-induced double-strand DNA Breaks. Mol Cell.

[41] Zou RS (2022). Massively parallel genomic perturbations with multi-target CRISPR interrogates Cas9 activity and DNA repair at endogenous sites. Nat Cell Biol.

[42] Chen X (2016). Manta: rapid detection of structural variants and indels for germline and cancer sequencing applications. Bioinformatics.

[43] Pannunzio NR (2018). Nonhomologous DNA end-joining for repair of DNA double-strand breaks. J Biol Chem.

[44] Patterson-Fortin J, D’Andrea AD (2020). Exploiting the microhomology-mediated end-joining pathway in cancer therapy. Cancer Res.

[45] Suzuki K (2003). Radiation-induced DNA damage and delayed induced genomic instability. Oncogene.

[46] Puck TT, Marcus PI (1956). Action of x-rays on mammalian cells. J Exp Med.

[47] Cosper PF (2022). Chromosome missegregation as a modulator of radiation sensitivity. Semin Radiat Oncol.

[48] Burgio G, Teboul L (2020). Anticipating and identifying collateral damage in genome editing. Trends Genet.

[49] Berkovich E (2007). Roles of ATM and NBS1 in chromatin structure modulation and DNA double-strand break repair. Nat Cell Biol.

[50] Goldstein M (2013). Nucleolin mediates nucleosome disruption critical for DNA double-strand break repair. Proc Natl Acad Sci U S A.

[51] Falco M (2023). Radiotherapy in pancreatic cancer: to whom, when, and how?. Cancers (Basel).

[52] Payton M (2024). Small-molecule inhibition of kinesin KIF18A reveals a mitotic vulnerability enriched in chromosomally unstable cancers. Nat Cancer.

[53] Chen Q (2024). Exploration of inhibitors targeting KIF18A with ploidy-specific lethality. Drug Discov Today.

[54] Al-Rawi DH (2024). Targeting chromosomal instability in patients with cancer. Nat Rev Clin Oncol.

[55] Marquis C (2021). Chromosomally unstable tumor cells specifically require KIF18A for proliferation. Nat Commun.

[56] Dewhurst SM (2020). Chromothripsis and telomere crisis: engines of genome instability. Curr Opin Genet Dev.

[57] Davoli T, de Lange T (2012). Telomere-driven tetraploidization occurs in human cells undergoing crisis and promotes transformation of mouse cells. Cancer Cell.

[58] Bae S (2014). Microhomology-based choice of Cas9 nuclease target sites. Nat Methods.

[59] Yuan B (2024). Modulation of the microhomology-mediated end joining pathway suppresses large deletions and enhances homology-directed repair following CRISPR-Cas9-induced DNA breaks. BMC Biol.

[60] Owens DDG (2019). Microhomologies are prevalent at Cas9-induced larger deletions. Nucleic Acids Res.

[61] Liu J (2022). Polyploid giant cancer cells: An emerging new field of cancer biology. Semin Cancer Biol.

[62] Moein S (2020). Cancer regeneration: Polyploid cells are the key drivers of tumor progression. Biochim Biophys Acta Rev Cancer.

[63] Coward J, Harding A (2014). Size does matter: why polyploid tumor cells are critical drug targets in the war on cancer. Front Oncol.

[64] Mahmoud M (2019). Structural variant calling: the long and the short of it. Genome Biol.

[65] Bowland K (2024). Islands of genomic stability in the face of genetically unstable metastatic cancer. PLoS One.

[66] Bulcha JT (2021). Viral vector platforms within the gene therapy landscape. Signal Transduct Target Ther.

[67] Butt MH (2022). Appraisal for the potential of viral and nonviral vectors in gene therapy: A Review. Genes (Basel).

[68] Kachanov A (2024). The menace of severe adverse events and deaths associated with viral gene therapy and its potential solution. Med Res Rev.

[69] Fontana M (2024). CRISPR-Cas9 gene editing with nexiguran ziclumeran for ATTR cardiomyopathy. N Engl J Med.

[70] Cohn DM (2025). CRISPR-based therapy for hereditary angioedema. N Engl J Med.

[71] Longhurst Hilary J (2024). CRISPR-Cas9 in vivo gene editing of *KLKB1* for hereditary angioedema. N Engl J Med.

[72] Dreaden EC (2018). RNA-Peptide nanoplexes drug DNA damage pathways in high-grade serous ovarian tumors. Bioeng Transl Med.

[73] Isaac I (2025). Reengineering endogenous targeting lipid nanoparticles (ENDO) for systemic delivery of mRNA to pancreas. Adv Mater.

[74] Mandalawatta HP (2024). Emerging trends in virus and virus-like particle gene therapy delivery to the brain. Mol Ther Nucleic Acids.

[75] Oieni J (2021). Nano-ghosts: novel biomimetic nano-vesicles for the delivery of antisense oligonucleotides. J Control Release.

[76] Tsilimigras DI (2021). Liver metastases. Nat Rev Dis Primers.

[79] Joung J (2017). Genome-scale CRISPR-Cas9 knockout and transcriptional activation screening. Nat Protoc.

[80] Li W (2014). MAGeCK enables robust identification of essential genes from genome-scale CRISPR/Cas9 knockout screens. Genome Biol.

